# Treatment Outcomes of Vaginal Infections on Sexual Function

**DOI:** 10.25122/jml-2020-0051

**Published:** 2020

**Authors:** Fatemeh Alahverdi, Masoomeh Kheirkhah, Leila Janani

**Affiliations:** 1. Department of Midwifery, Iran University of Medical Sciences, Tehran, Iran; 2. Nursing Care Research Center (NCRC), Department of Reproductive Health and Midwifery, School of Nursing and Midwifery,Iran University of Medical Sciences, Tehran, Iran; 3. Department of Biostatistics, Iran University of Medical Sciences, Tehran, Iran

**Keywords:** Treatment, outcome, sexual function, vaginal infection

## Abstract

Vaginal infections are the most common gynecological diseases and one of the causes of sexual dysfunction. In more than 50% of patients, sexual dysfunction is twice as common. Evaluation of the treatment outcome of vaginal infections can be effective in identifying factors related to the sexual function. This is a descriptive-observational study that included patients referred to Imam Sajjad Shahriar Hospital during 2017-2018. Patients that met the inclusion criteria (with vaginal infections of Candida, Gardnerella and mixed infections - Candida and Gardnerella - Mixed group) completed the Female Sexual Function Index (FSFI) questionnaire before and one month after treatment. Data were analyzed by SPSS 16, paired t-test, ANOVA, and multiple regression. P-value <0.05 was considered significant. After the treatment of vaginitis, there was a significant increase in all aspects of the FSFI questionnaire. The mean and standard deviation of sexual function of women before and after treatment were 18.26 ± 4.36 and 26.27 ± 4.97 in the Candida group, 20.06 ± 4.94 and 25.87 ± 5.32 in the Gardnerella group, and 19.69 ± 4.25 and 27.05 ± 5.12 in the Mixed group. Prior to treatment in the Mixed and Gardnerella group, the most important sexual dysfunction was related to the dyspareunia component, while in the Candida group, the most important sexual dysfunction was related to the desire component. After treatment, the components of dyspareunia in the Mixed and Gardnerella group and the orgasmic component in the Candida group showed the greatest improvement. The regression test showed that the effect of age, body mass index and duration of sex on sexual function was significant (P <0.05). Duration of the disease had the greatest impact on sexual dysfunction, and after treatment of the disease, sexual function improved significantly. Proper diagnosis and treatment are effective in improving women’s sexual function. The results of this study can be promoted to midwives and gynecologists.

## Introduction

Sexual relationships shape the perceptions and continuity of couples’ lives [1]. One-third of women do not enjoy their sexual intercourse and one-quarter of them do not reach the peak of sexual pleasure. For many women, sexual dysfunction is physically and psychologically disturbing [2]. The worldwide prevalence of sexual dysfunction is 40% [3] and the corresponding rate in Iran is 43% [4]. Genitourinary tract disorders can affect sexual dysfunction [5]. Sexual dysfunction is the most common complaint in women with pain, slippage, and other medical problems [6]. Less frequent sexual intercourse may lead to inactive sexual life and decrease couples’ sexual satisfaction [7].

The prevalence of women with vaginal infections that have sexual dysfunction is 53% [8]. Recent studies have reported a prevalence of vaginal infections in children of reproductive age of 13.4-21%. The prevalence of vaginitis in the age group of 25-29 years is 7.8%, in the age group of 30-34 years is 10.5%, in the age group of 35-39 years is 11.1%, and13.8% in the 40-44 age group [9]. Candida albicans, trichomonas vaginitis, and bacterial vaginitis are the most common causes of vaginitis and are responsible for almost 80% of referrals to women’s clinics [10, 11]. In more than 50% of patients with vulvovaginitis, sexual dysfunction is twice more common than healthy women [8]. Women with vulvovaginitis have mild sexual dysfunction compared to those with vulvovaginal deformities (including vestibulitis, lichen sclerosus, and atrophic vaginitis), and scored lower on dyspareunia and slippage [8]. Decreased sexual desire and satisfaction have been reported in some patients with recurrent vulvovaginal candidiasis [12]. Sexual dysfunction is one of the most important causes of divorce and sexual dissatisfaction was reported in 66.7% of men and 68.4% of women [13]. Besides, according to divorce statistics in Tehran (the capital of Iran), one divorce occurs for every four marriages [14]. This study is significant as it provides some insights for vaginal treatment and improving sexual dysfunction. There is no study in the literature to provide a comparison of sexual function before and after the treatment of vaginal infections. This gap in the literature motivated this line research. Given the role of midwives in counseling, diagnosing, and treating gynecological infections, the results of this study can provide insights to be used by midwives, gynecologists, and women health planners. 

## Material and Methods

**Figure 1:**
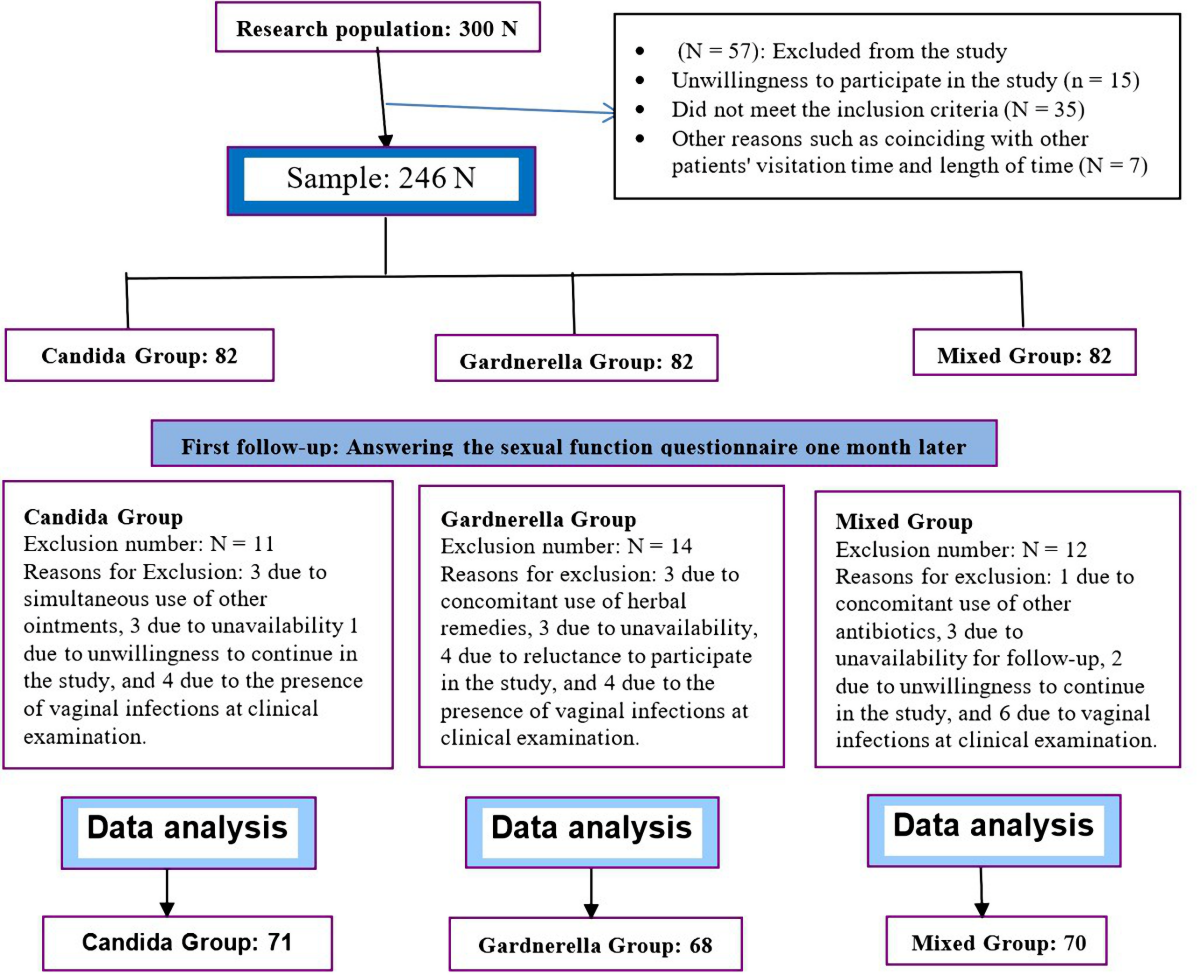
Study method diagram.

The present study is an uncontrolled before-after study that was performed to determine the effects of treatment of vaginal infections on the sexual function in women referred to the Imam Sajjad Hospital from October 2016 to June 2017. After obtaining the permission and approval of the Ethics Committee (IR. IUMS. REC No. 1396. 9411373001) of Iran University of Medical Sciences and after receiving a letter of introduction, the researcher was referred to the Imam Sajjad Hospital. The study diagram is presented in Figure 1. Informed consent was obtained from the patients infected with Candida, Gardnerella, or who had mixed infections (Candida and Gardnerella, trichomonas vaginalis and other possible pathogenic causes of vaginal infections) whose disease was confirmed via clinical examination and were willing to participate in the study. The inclusion criteria were the following: having a vaginal infection (with all possible vaginal infection pathogenic causes), being literate, women of reproductive age (15-45 years), husband’s monogamy, a body mass index of less than 30, taking no drugs, having no chronic diseases such as diabetes, hypertension, and heart disease, not breastfeeding, the absence of menopausal complications and mental illnesses in the participant and her husband (as was stated by the patient), and the absence of any lesion affecting sexual function in the genital area. The exclusion criteria include migration and unavailability of patients for follow-up, as well as failure to use the drug more than twice a week (according to the patient), and the concomitant use of other drugs to treat vaginal infections during the study. The following guidelines were followed for diagnosis of infections: 

Candida vaginitis was confirmed and diagnosed by direct observation of a white, cottage cheese-like discharge, pH <4.5 on a pH paper, vulvar and vaginal erythema, and the diagnosis confirmation from a gynecology clinic. Vaginitis due to Gardnerella infection was diagnosed when three of the following four criteria were met: homogeneous gray discharge, fish odor before or after adding 10% potassium hydroxide (Whiff test positive, fish odor), pH> 4.5, and Key cells in microscopic examination. Mixed vaginitis was also confirmed upon the diagnosis of both Candida and Gardnerella infections mentioned above. Finally, the patients were treated according to the protocol prescribed by a gynecologist. 

The patients with fungal infection were treated with clotrimazole (1% vaginal cream for 7 nights and topical clotrimazole ointment 2 times daily for 7 days). The patients with bacterial vaginitis were treated with metronidazole (250-mg tablets every 12 hours for 7 days), and the patients with mixed infections were treated with clotrimazole (1% vaginal cream for 7 nights and topical clotrimazole (ointment 2 times daily for 7 days) and metronidazole (250-mg tablets every 12 hours 2 for 7 days). 

The participants were selected through purposive sampling in this study. Initially, as there was no information on the mean and variance of sexual function score in three groups of (Candida, Gardnerella, and Mixed), a pilot study was performed on 30 patients (10 persons in each group) and the FSFI questionnaire was completed by the patients referred to the Imam Sajjad Shahriar Hospital. Based on the pilot data, the maximum standard deviation for the sexual function score was 9 in the Candida group. Considering a 4-unit difference in sexual performance score in this group before and after the treatment and assuming a 50% correlation between the pre- and post-test scores, for the 80% power and 5% error probability level, 73 people were sufficient in this group. 

The data collection tools were the demographic and clinical information form and the Persian version of a sexual function questionnaire that was completed by the eligible patients before starting the treatment. The reliability of the sexual function questionnaire was reported by Rosen to be equal to 0.82 [21]. In this study, the validity of the questionnaire was determined by the content validity method, and its reliability by Cronbach’s alpha was 0.87. The Sexual Function Questionnaire contains 19 items grouped in six different domains that constitute the totality of sexual function, including desire, satisfaction, stimulation, orgasm, slippage, and dyspareunia. A score lower than 9.3 for each of the six components of total sexual function was considered to show impaired function and a score equal to or higher than 9.3 indicated a normal function. 

One month after treatment, the patients were contacted and asked to return to the women’s clinic for re-examination and completing the questionnaires. After re-examination and assurance of improvement, the sexual function questionnaire was completed by the patients. The collected data were analyzed by SPSS 16 using paired samples t-test, ANOVA, and multiple regression at the significance level of 0.05.

## Results

Table 1 shows that the mean scores of the sexual function before and after the intervention increased significantly for the members of all groups (p <0.05). The mean increase in the post-test was 5.86 for the Mixed group, 6.51 for the Candida group, and 4.16 for the patients in the Gardnerella group. The mean pre-test scores of sexual function were significantly different in the three groups (p <0.008). The mean scores of sexual function were 18.26 ± 4.36, 20.06 ± 4.94, and 19.69 ± 4.25 for the participants in the Candida group, the Gardnerella group, and the Mixed group, respectively. However, the post-treatment sexual function scores were not significantly different between the three groups (p = 0. 096). 

**Table 1:** Sexual function by type of group and test stage.

Variables Groups		Statistics	Pre-test	Post-test	p-value Paired t	p-value Pre-test	p-value Post-test
**Sexual function**	**Mixed** (70=n)	Mean SD	19.69 4.25	27.05 5.12	0.001 >	0.008 >	0.096>
	**Candida** (71=n)	Mean SD	18.26 4.36	26.27 4.97	0.001 >		
	**Gardnerella** (68=n)	Mean SD	20.06 4.94	25.87 5.32	0.001 >		

As can be seen in Table 2, there is a significant increase in the mean pre-test and post-test scores of 6 components of sexual function in all groups after the treatment (p <0.05), indicating significant improvements in all components of sexual function in all three groups after the treatment. ANOVA was performed to compare the post-test scores of the three groups, and the results showed that the treatment was not effective for the participants’ sexual desire and orgasm. However, it was effective for sexual stimulation, lubrication satisfaction, and dyspareunia. 

**Table 2:** The components of sexual function by type of group and stage of the test.

Components Group		Statistics	Pre-test	Post-test	p-value Paired t	p-value Post-test
**Desire**	**Mixed** (70=n)	Mean SD	2.87 0.89	4.13 1.12	0.001 >	0.373
	**Candida** (71=n)	Mean SD	2.57 0.59	4.27 0.95	0.001 >	
	**Gardnerella** (68=n)	Mean SD	2.97 0.66	4.12 0.83	0.001>	
**Sexual arousal**	**Mixed** (70=n)	Mean SD	2.98 0.57	3.93 0.87	0.001 >	0.013
	**Candida** (71=n)	Mean SD	3.20 0.64	4.45 1.26	0.001 >
	**Gardnerella** (68=n)	Mean SD	3.17 0.70	4.10 1.17	0.001 >
**Lubrication**	**Mixed** (70=n)	Mean SD	3.68 0.91	4.72 1.20	0.001 >	0.001 >
	**Candida** (71=n)	Mean SD	3.14 0.84	4.32 1.10	0.001 >	
	**Gardnerella** (68=n)	Mean SD	3.35 0.76	3.97 0.95	0.003>	
**Orgasm**	**Mixed** (70=n)	Mean SD	3.73 0.95	4.34 1.3	0.001 >	0.142
	**Candida** (71=n)	Mean SD	3.49 0.82	4.58 1.21	0.001 >
	**Gardnerella** (68=n)	Mean SD	3.78 0.85	4.49 1.13	0.001 >
**Sexual satisfaction**	**Mixed** (70=n)	Mean SD	3.59 0.70	4.88 0.93	0.001 >	0.004
	**Candida** (71=n)	Mean SD	3.10 0.93	4.20 0.84	0.001 >	
	**Gardnerella** (68=n)	Mean SD	3.84 0.61	4.59 1.16	0.001 >	
**Dyspareunia**	**Mixed** (70=n)	Mean SD	2.84 0.72	5.1 1.18	0.001 >	0.011 >
	**Candida** (71=n)	Mean SD	2.76 0.93	4.45 1.28	0.001 >
	**Gardnerella** (68=n)	Mean SD	2.95 0.86	4.60 1.14	0.001 >

**Figure 2:**
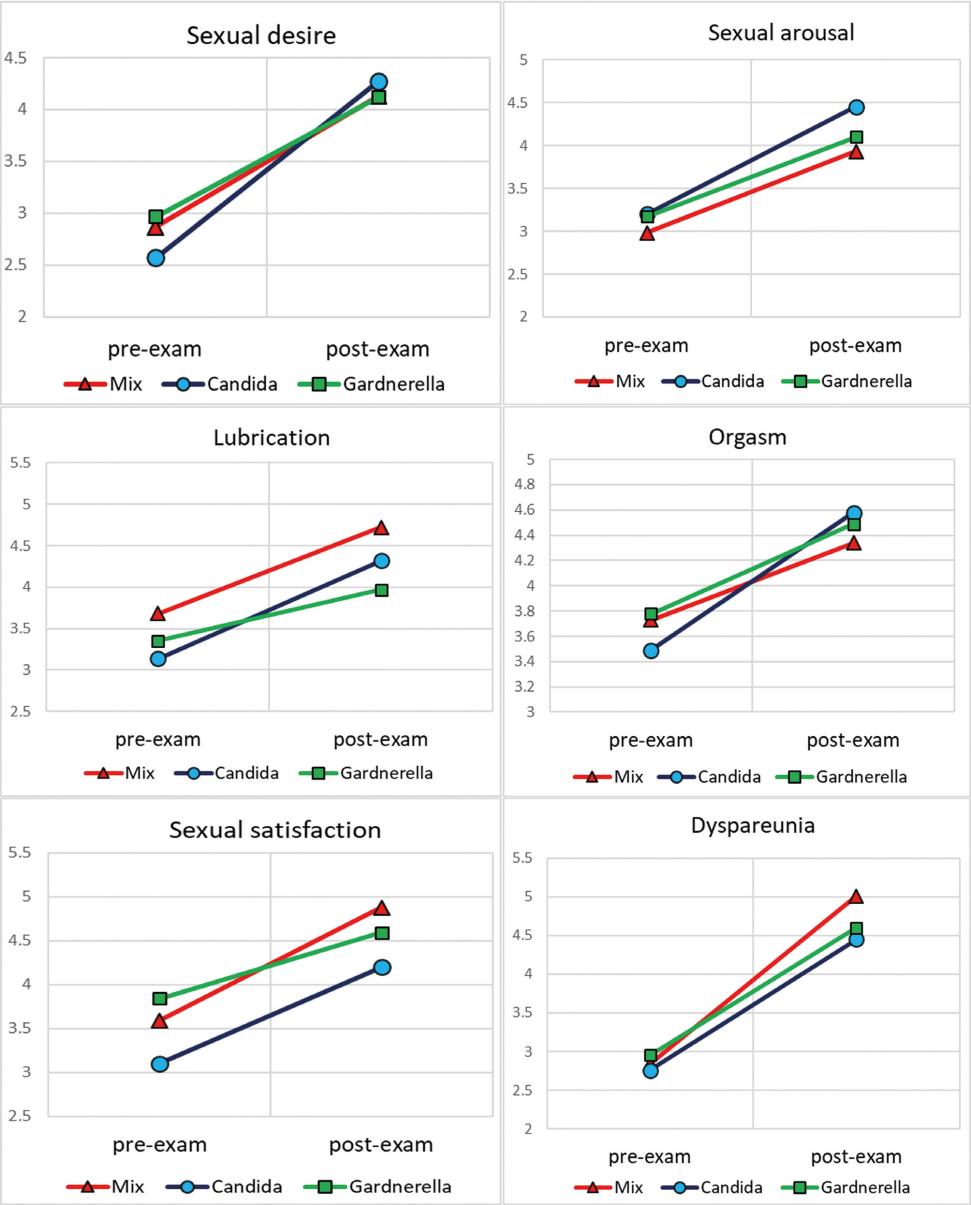
Linear graphs of mean sexual performance components by groups and test stages.

As shown in Table 3, the multiple regression test with the Enter method was used to investigate the effect of demographic variables (education, age, body mass index, and duration of disease) on sexual function. The results showed that age, body mass index and duration of sex were effective on sexual function (p <0.05). The highest effect on sexual function was related to the duration of infection with a coefficient of -0.298. The linear diagrams of the components (Figure 2) show an increase in the post-test scores in all groups.

**Table 3:** Regression tests with the aim of predicting sexual performance based on demographic variables.

	Independent variable	Non-standard coefficient	Standard error	Standard coefficient	T statistics	P-value	Coefficient
**Sexual function**	Education	0.166	0.652	0.020	0.255	0.799	0.32
	Age	-0.284	0.079	-0.278	-3.59	0.001>	
	BMI	-0.524	0.198	-0.241	-2.65	0.009>	
	Duration of disease	-0.904	0.272	-0.298	-3.32	0.001>	

## Discussion

The main purpose of this study was to determine the effects of the treatment of vaginal infections on sexual function. 

The results of the study indicated that the overall score of sexual function in the Mixed group was higher than the other two groups after the treatment. In the Mixed and Gardnerella groups, dyspareunia showed the greatest improvement, while orgasm showed the biggest improvement after the treatment in the Candida group. The patients in all three groups scored higher than 3.9 on all dimensions of the questionnaire. In the present study, the effect of Candida infection treatment on orgasm, dyspareunia, and sexual arousal was more significant than the other components. The results of the study of Costantini et al. on the sexual function of women after pelvic organ prolapse treatment were similar to the findings of the present study. Moreover, the patients showed the greatest improvement after surgery and repair of the anterior and posterior vaginal wall prolapse in terms of orgasm, dyspareunia, and sexual arousal. Accordingly, it can be concluded that the treatment of diseases affecting the genital area can have an impact on sexual arousal and reduce painful intercourse. The present study also showed that after the treatment of vaginal infections and dyspareunia in the Mixed and Gardnerella groups had the greatest improvement regarding the women’s sexual function. 

A study by Arslan et al. on the treatment outcomes of bladder pain syndrome (bladder inflammation) on female sexual function showed that patients had sexual dysfunction before and after the treatment and remission of symptoms such as burning and discharge, and showed improvement in all aspects of female sexual function, including dyspareunia. The results of this study were consistent with the present study, and it can be concluded that the timely treatment of urogenital diseases can improve sexual function. The patients in this study complained about vaginal infection symptoms during sexual intercourse and were satisfied with their sexual function after the treatment. 

The results of Hor et al. ’s study showed remission of abdominal pain symptoms, decreased mucosal secretions during proximity, less painful intercourse and significant improvement in all aspects of sexual function in patients with ileal anastomosis. The most significant improvement was related to dyspareunia and satisfaction. 

These results were in agreement with the results of the present study concerning all three types of vaginitis and it can be concluded that the treatment of urogenital and anal diseases can be effective in all aspects of the sexual function of women, especially regarding dyspareunia, eliminating unhealthy sexual intercourse. The present study showed a positive relationship between dyspareunia and other components of sexual function after the treatment. With the improvement of the pain component, the scores of other components also increased proportionally. 

Bastani et al. examined the effect of physical pelvic floor treatments on painful intercourse in women and showed that all sexual function components increased after treatment. Besides, there was a positive correlation between all the components and dyspareunia before and after the treatment, as shown in the present study. 

The result of this study showed that age, body mass index and duration of infection had a significant effect on sexual function. Dyspareunia had the greatest improvement after the treatment in the Mixed and Gardnerella groups. The participants’ age was correlated with sexual desire, stimulation, and lubrication.

In line with the results of the present study, Mazinani et al. and Botros also showed that with increasing age, the sexual function of women decreases.

Esposito et al. ’s study, which compared women with and without sexual dysfunction, found that the mean score of female sexual function was correlated with body mass index, sexual arousal, vaginal moisture, and orgasm. They also showed that sexual satisfaction in obese individuals was significantly lower compared to persons with a normal weight. Sexual dysfunction accompanied obesity, and the effects of weight gain appeared in terms of sexual dysfunction. 

The present study showed the patients with vaginitis with body mass index higher than 25 had a lower sexual function and there was a significant relationship between body mass index with sexual desire, vaginal wetness, orgasm, and sexual satisfaction. 

### Limitations of the study

The Sexual Function Questionnaire examines sexual performance in the past month. One of the limitations of this study may be that some participants in the research samples did not have intercourse within 4 weeks after the treatment, which was outside the control of the researcher. To overcome this limitation, the researcher provided the participants with the necessary training at baseline. Besides, to track and prevent the dropout of the participants, the researcher recorded their phone numbers to know when the patients were coming to the clinic. Moreover, the costs of the patients’ visits to the clinic and their travel expenses were paid. Breast screening was performed free of charge for all participants to encourage them to continue their participation.

## Conclusion

Sexual dysfunction was associated with decreased sexual function, marital satisfaction, vaginal infections, and reduced self-esteem. 

Therefore, prompt treatment of the disease can improve symptoms faster. Recent research has shown the effectiveness of treatment on increasing women’s sexual function. Considering the responsibilities of midwives in screening, personal health education, and other aspects of the service for women at the childbearing age, the use of inexpensive and affordable therapies is recommended according to the vaginal infection treatment protocol. 

## Acknowledgments

This study was part of a master’s thesis in Midwifery Education at Iran University of Medical Sciences. This work was supported by the Iran University of Medical Sciences grant number [4891-9411373001.

## Conflict of Interest

The authors declare that there is no conflict of interest.
